# The importance of validated alpha taxonomy for phylogenetic and DNA barcoding studies: a comment on species identification of pygmy grasshoppers (Orthoptera, Tetrigidae)

**DOI:** 10.3897/zookeys.679.12507

**Published:** 2017-06-12

**Authors:** Arne W. Lehmann, Hendrik Devriese, Josef Tumbrinck, Josip Skejo, Gerlind U.C. Lehmann, Axel Hochkirch

**Affiliations:** 1 SIGTET-Special Interest Group Tetrigidae, http://home.scarlet.be/~ping0646/index.html; 2 Friedensallee 37, 14532 Stahnsdorf, Germany; 3 Koninklijk Belgisch Instituut voor Natuurwetenschappen, Direction of Taxonomy and Phylogeny, Vautierstraat 29, 1040 Brussel, Belgium; 4 Auf der Hees 1, 41849 Wassenberg, Germany; 5 University of Zagreb, Faculty of Science, Department of Biology, Rooseveltov trg 6, HR-10000 Zagreb, Croatia; 6 Humboldt University Berlin, Department of Biology, Invalidenstrasse 43, 10115 Berlin, Germany; 7 Trier University, Department of Biogeography, 54286 Trier, Germany

**Keywords:** Orthoptera, Tetrigidae, Barcoding, taxonomy, misidentification

## Abstract

In a recently published paper on colour polymorphism in a Pygmy grasshopper from China (Zhao et al 2016) an unidentified *Paratettix* sp. was misidentified as *Tetrix bolivari*. This case highlights the need for correct species identification and provides an opportunity to recommend some aspects of Good Taxonomic Practice (GTP) in Tetrigidae to reduce the number of erroneous identifications.

## Introduction

For a number of reasons, nobody can avoid misidentifications of insect species. However, verified species identification is a backbone for many biological disciplines. Among Orthoptera, the members of the family Tetrigidae have a quite complicated taxonomic history, including long list of synonyms and large collections of unidentified specimens. In Europe, it took almost 200 years, from Linnean time to the publication of the European handbook by Harz (1975), to provide reasonable taxonomic stability. With increasing speed of taxonomic research in other regions of the world, we observe similar problems of taxonomic inflation. With respect to Tetrigidae taxonomy, researchers from China are currently particularly active, having described 795 species (compare Cigliano et al. 2017). While we applaud the effort of our Chinese colleagues, we observe some problems with interpretation of well-known Palaearctic species.

In a recent publication in ZooKeys, Zhao et al. (2016) have shown that genetic haplotype diversity of the Barcoding locus COI is incongruent with colour polymorphism found in a Chinese pygmy grasshopper (Tetrigidae). While the molecular data by Zhao et al. (2016) of 57 individuals supports the hypothesis that the colour morphs studied by them all belong to a single species, we have evidence against the identification of the species. A great benefit of the publication by Zhao et al. (2016) is that it not only includes COI barcodes, but also presents photos of a number of specimens. This allows us to reflect on the identity of the species under study. We first provide some evidence that the species depicted in the publication of Zhao et al. (2016) is misidentified. For clarification, we outline the known distribution of *Tetrix bolivari*, which is unlikely to occur in China and recommend some aspects of Good Taxonomic Practice (GTP) in Tetrigidae.

The photos of several individuals presented by Zhao et al. (2016) in their figure 3 show individuals belonging to the genus *Paratettix* Bolívar, 1887. This is evident from the traits visible (see for example Devriese (1999) and Tumbrinck (2014) for detailed descriptions of useful morphological characters in Tetrigidae). (1) The Median Carina (CM) of the pronotum terminates before reaching the distal pronotal end, (2) the interocular distance is as broad as the eye-span, (3) the fastigium does not protrude in front of the eyes and (4) the long antennae segments slightly broaden towards their terminal end. The first character is very distinctive and the single-most important character aligning the specimens to the genus *Paratettix*, as outlined in the detailed keys for Europe (Harz 1975) and sub-Saharan Africa (Günther 1979). Interestingly, these characters are also referred to in a key presented by three Chinese authors (Deng et al. 2007) in which the senior author of Zhao et al. (2016) is involved. Using their key (Deng et al. 2007, p. 422) to identify the specimens shown in their figure 3 we end up with the genus *Paratettix*. This genus has a global distribution and occurs mainly in the tropics, including many extremely common species in open tropical lowland habitats (e.g. Günther 1979). The quality of the photos does not allow a proper identification to the species level. Without material at hand, our best guess is a species identical with or close to *Paratettix obesus* Bolívar, 1887 (compare Tumbrinck 2015).

**Figure 1.**
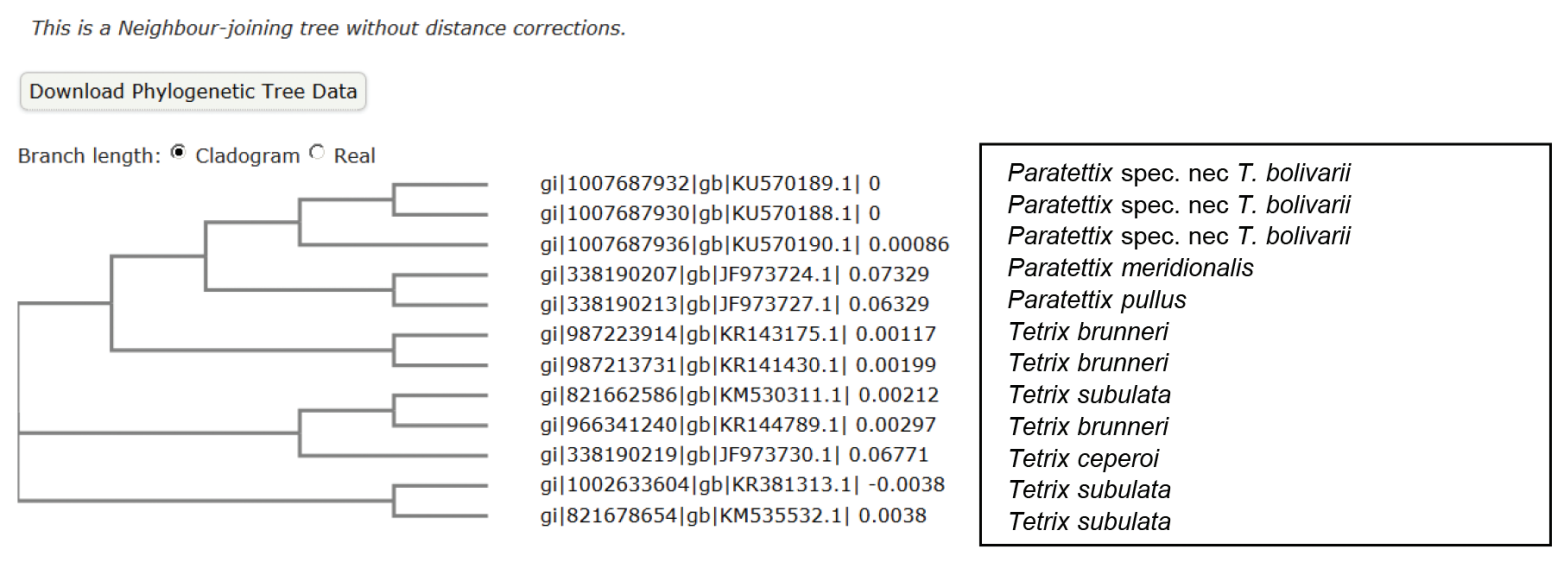
Neighbour-joining tree based upon the COI data, illustrating the clustering of the Chinese samples by Zhao et al. (2016) together with *Paratettix* and not *Tetrix* species, data extracted from the Barcoding of life project.

A second line of evidence comes from genetic data. The authors sequenced the gene COI (which is commonly used as Barcoding gene) to examine a proposed correlation between colour morphs and haplotypes. As sequences of COI are available for a wide range of taxa, we deployed a quick analysis of the mitochondrial COI data from the Barcoding of life database (BOLD version 3; Ratnasingham and Hebert 2007) in conjunction with some of the Chinese data by Zhao et al. (2016). We chose gene sequences from a selected subset of species belonging either to the genus *Tetrix* or *Paratettix*, together with three sequences from the study of Zhao et al. (2016). Detailed information on the determination and sequencing history can be found in the BOLD system (www.barcodinglife.org). A neighbour-joining tree was produced running the software MUSCLE (Edgar 2004) online on the EBI Server (www.ebi.ac.uk/Tools/msa/muscle/). The resulting tree (Figure 1) is in line with the morphological evidence, as the Chinese individuals build a monophyletic cluster with *Paratettix meridionalis* (Rambur, [1838]) and *Paratettix pullus* Bolívar, 1887, rather than to group with selected members of the genus *Tetrix* Latreille, [1802]. It should be noted that the gene COI contains a reasonable phylogenetic signal for a smaller number of species of moderate separation time, and was shown to be quite useful for Barcoding purposes in Orthoptera as well (Hawlitschek et al. 2016).

We can add a biogeographic argument, why we question the occurrence of *Tetrix bolivari* in China in general. This species has been studied in detail for ecological (Kočárek et al. 2011) and faunistic reasons. *Tetrix bolivari* is distributed from southern Europe (Devriese 1996), the Middle East (Lehmann and Monnerat 2014) to Central Asia, where it is found in Turkmenistan (Podgornaya 1983; Storozhenko et al. 1994) and Tadzhikistan (Harz 1979). The tropical and subtropical regions of China have a very distinct fauna, making the occurrence of a Mediterranean-Central-Asian species unlikely. We therefore encourage our Chinese colleagues to re-examine their material. Interestingly, when adding 12S rDNA sequence data published independently under the name *Tetrix bolivari* from China (Chen and Jiang 2004) to our own alignment of Palaearctic Tetrigidae (Hochkirch et al., in prep.), they cluster with the widespread East-Asian species *Tetrix japonica* (Bolívar, 1887). In summary, both testable records of *Tetrix bolivari* from China appear to belong to other species.

We summarize some recommendations for a Good Taxonomic Practice (GTP) to reduce the taxonomic instability in Tetrigidae:

A fundamental aspect of morphological work on Tetrigidae should be a common terminology of the body parts. A large set of morphological traits are depicted and named by Devriese (1996, 1999) and recently by Tumbrinck (2014). We strongly recommend using this terminology for an easier comparison of characters.

An astonishing but common mistake in Tetrigidae identification is the misinterpretation of nymphs for adults. Tetrigidae can show interesting phenologies, including cohort splitting between years. This phenomenon is quite well studied in European species (e.g. Schulte 2003, Pushkar 2009) such phenomena can also be expected in the tropics, less so in lowlands but especially on mountains. Especially in species with unknown adult phenology the collection of individuals from the same locality over the season might reduce this problem.

Along with studies on the morphology we need a better understanding of phenotypic variation between individuals. Such phenotypic data of adults have been shown to be helpful to reduce the instability in hard to distinguish *Paratettix* species from Africa (Günther 1979).

Tetrigidae have a tendency to exhibit extensive morphological polymorphisms, such as different wing morphs. Wing dimorphism is best studied and understood in *Tetrix subulata* (e.g. Steenman et al. 2013, 2015), and a frequent phenomenon in European (summarized in Devriese 1996) and African species (Günther 1979). The easiest way to test for wing dimorphism in Tetrigidae is obviously to study larger sample sizes and obtain data on body and hind wing dimensions. No such data seem to exist to date for Asian species, but we would not be surprised if some of the newly described species from China would turn out to represent wing morphs of previously described species.

A further striking polymorphism in Tetrigidae is found in body colouration. Colour polymorphism is widespread within Tetrigidae species, even within the same populations (e.g. Nabours 1930; Hochkirch et al. 2007, 2008), see also the current study by Zhao et al. (2016). From the beginning of Tetrigidae taxonomy, colour variation has caused taxonomic uncertainties until it was recognized as an intraspecific phenomenon, see e.g. the list of synonyms for *Tetrix subulata* in Harz (1975). We have to assume that a number of newly described Tetrigidae species from China merely represent colour morphs rather than new species.

Until improved determination keys (using multiple characters being complemented by drawings or photographs) along with verified molecular data are available, showing photographs of the studied specimens is a helpful practice. It was of great benefit that the article by Zhao et al. (2016) provided photos of specimens in combination with gene sequences, which allowed us to reflect on the species identity.
